# Individual and contextual factors related to dental caries in underprivileged Brazilian adolescents

**DOI:** 10.1186/1472-6831-15-6

**Published:** 2015-01-20

**Authors:** Fabiana de Lima Vazquez, Karine Laura Cortellazzi, Armando Koichiro Kaieda, Jaqueline Vilela Bulgareli, Fabio Luiz Mialhe, Glaucia Maria Bovi Ambrosano, Elaine Pereira da Silva Tagliaferro, Luciane Miranda Guerra, Marcelo de Castro Meneghim, Antonio Carlos Pereira

**Affiliations:** Department of Community Dentistry, Piracicaba Dental School, State University of Campinas, São Paulo, Brazil, Av. Limeira 901, P.O. BOX 52, CEP: 13414-903 Piracicaba, SP Brazil; Department of Community Dentistry, Araraquara Dental School, State University of São Paulo, São Paulo, Brazil, Rua Humaitá, 1680, Araraquara, SP 14801-903 Brazil

**Keywords:** Dental caries, Risk assessment, Social vulnerability, Adolescent behavior

## Abstract

**Background:**

Investigate the individual and contextual variables related to caries in underprivileged adolescents, and the disparity in distribution of the disease.

**Methods:**

Cross-sectional analytical study, conducted in the city of Piracicaba, SP, Brazil, in 2012. The probabilistic sample was composed of 1,179 adolescents from 15–19 years of age, randomly selected from 21 state schools and 34 Primary Health Units – Family Health (PHU-FH). The dependent variables studied were number of decayed teeth and caries experience (DMFT). The independent variables were classified into individual (clinical, sociodemographic, psychosocial, self-perception, impact on oral health, access to services, and quality of life) and contextual (social exclusion index, total number of residents in suburb, literacy rate, and the following variables given in percentages: residences in the home ownership category, provision of domestic sewerage, trash collection, families with income of over 1 minimum wage per month, and families without monthly income) variables. The multilevel regression model was estimated by the PROC GLIMMIX (Generalized Linear Models-Mixed) procedure, considering the individual variables as Level 1 and the contextual variables of the suburbs as Level 2. Adjustment of the model was evaluated by -2 Res Log Likelihood with α = 0.05.

**Results:**

As regards the individual variables, adolescents who declared having a prison inmate in the Family and resided in homes with a larger number of persons, showed a higher number of decayed teeth. There were a larger number of decayed teeth, a higher DMFT value, and worse self-perception as regards the health of their teeth and mouth. Other variables, such as being of the female gender, age and time since last visit to the dentist were related to the DMFT index. As regards the contextual variables, the DMFT was lower in suburbs with greater access to domestic sewage, and the number of decayed teeth was higher in suburbs with the worst social exclusion indices.

**Conclusion:**

Individual and contextual variables were associated with the presence of caries and DMFT index in underprivileged adolescents, indicating that they must be taken into consideration in the formulation of policies directed towards oral health promotion and prevention activities in this group.

## Background

Over the last few decades, a change in the epidemiological profile of dental caries has been observed in developed [1] and developing countries. In Brazil, the same trend has been verified, especially in children and adolescents, with a significant reduction of 35% in the carious component being observed in adolescents between the years 2003 and 2010 [2].

However, this qualitative improvement in the Brazilian Population’s level of oral health has been accompanied by a change in the distribution of the disease, with it being concentrated to a larger extent in the socio-economically vulnerable groups [3].

Theories have been developed to explain the relationship between socioeconomic status (SES) and health, with cultural, behavioral, material, structural and psychosocial factors being modulators of this relationship [4–6]. Therefore, not only do they study the relationship between SES and health, poverty and healthy behaviors, but these theories seek an explanation for causal pathways of oral health outcomes, risk behaviors and use of oral health services [7, 8].

Although the inequalities in the prevalence of caries in adolescents for the different social gradients have been observed in some studies [9–12], the contribution of contextual factors for the risk of disease still require further studies. This is particularly necessary with respect to underprivileged populations, in order to elucidate the differences within the social environment [13, 14], and thus justifies new studies [12]. Similarly, when there is interest in identifying small areas with high levels of need for dental treatment [15], vulnerability variables can be useful because they are sensitive to variations in oral health and oral health behaviors.

Brazil appears to be an ideal country for conducting epidemiological studies for the evaluation of inequalities in health, considering that at the same time as it presents remarkable social inequality, changes are seen in economic growth, socioeconomic gradients and improvement in the GINI (distribution of income), especially over the last decade [16].

Therefore, it is important to know the impact of social determinants on health, both at individual and collective level, in order to plan actions at a local level [17]. However, for the adolescent population, little has been investigated about the relationship between social inequalities and oral health behaviors [8].

In addition to social factors, proximal factors such as self-perception of health have been associated with oral health and quality of life indicators [18, 19]. Self-perception of health is the subjective indicator most used in epidemiological researches, influencing health behaviors and being influenced by SES [8]. Therefore, it is an important component for measuring oral health status and must be taken into consideration in the elaboration of approaches to diminishing inequalities in health [20].

Analyses of risk factors for caries in Brazilian adolescents have pointed out that the following variables: past history of caries [21], activity of the disease, parents’ educational level [22] and socioeconomic factors [10, 11] were shown to be the most significant in statistical models. Therefore, in addition to identifying traditional risk factors, the present study, in an approach to the population of underprivileged adolescents, endeavored to find out the variables that identify environments of vulnerability, mainly connected with the individual and family (self-perception of health, reports of pain, access to the health system, prison inmate in the family, number of persons in the family, family cohesion, and others) and the contextual aspects (social exclusion index and other factors).

Nevertheless, adolescents constitute a group under constant exposure to other important factors, such as emotional, social, and physical situations, etc., which makes this analysis more complex. Although adolescence is a time when important psychosocial changes occur, which may put youngsters at increased risk for general and oral problems, we observed that few studies have demonstrated the impact of social determinants on oral health in adolescents [12, 23]. In addition, most of these studies were conducted on an individual basis; there are few that evaluate the impact of territorial characteristics on their oral health, especially when we study the underprivileged population. Therefore, identification of explanatory variables by means of using multilevel analysis may provide greater insight into the interrelationships between the two different levels of effects (individual and contextual), as well as insights into how these relationships affect dental caries. This technique provides better estimates and gives substantive meanings to data clustering in comparison with traditional regression analysis [24].

There are an increasingly higher number of risk factors that augment the probability of developing poor oral health and caries. However, it has not yet been determined which factors create the greatest risks. The general model of vulnerability considers those who experience multiple risks to be more vulnerable to poor outcomes than those with one or no risks. Vulnerability leaves the child unprotected, creating high probability of developing poor oral health, and when a child is exposed to risk factors within the family or community, this vulnerability increases and there is an even greater probability of a poorer dental health outcome [25].

In the light of this scenario, and with a view to better qualification of public health policies for this group in a stage of transition, the aim of this study was to identify the individual and contextual variables related to caries in underprivileged adolescents in the southeastern region of Brazil, and to study the disparity of distribution of the disease.

## Methods

### Ethical aspects

This study was approved by the Research Ethics Committee of FOP-UNICAMP, in accordance with resolution 196/96 of the National Health Council, Ministry of Health, under Protocol No.027/2011.

### Type of study

Analytical Cross-sectional Study.

### Study location

This study was developed in the Municipality of Piracicaba, SP, Brazil, in the period from January to December, 2012, with adolescents from 15 to 19 years-old, who were under the care of Primary Health Care- Family Health teams (PHC-FH), which provide primary health care for all family members residing in a circumscribed area (about 4,000 persons) [26]. They were enrolled in public schools (located in the territories covered by these PHC-FH units) and in the PHC-FC units. Social exclusion occurs to the greatest extent in these regions in the municipality.

The city has an estimated population of 368,843 inhabitants, a Human Development Index (HDI) of 0.84 and has had a fluoridated public water supply since 1971 (0.7 ppmF).

### Study universe

The city is composed of 68 suburbs distributed throughout 5 administrative regions (North, South, East, West and Center) and there are a total number of 12,539 adolescents in the age-range from 15 to 17 years. The Northern Region consists of 14 suburbs with 2,460 adolescents; Southern Region, 14 suburbs and 2,510 adolescents; Eastern Region, 16 suburbs and 2,491 adolescents; Western Region, 13 suburbs and 3,330 adolescents; Downtown Region, 11 suburbs and 1,748 adolescents.

According to data from the Municipal Secretary for Health, in 2012 there were a total number of 34 PHC-FH units, and among them there were 12 units with Primary Dental Care (PDC) teams. On an average, 320 adolescents between the ages of 15 and 19 years were enrolled in each one of the PHU-FH unit, totaling approximately 11,000 individuals. According to the Secretary for Education, the municipality had 43 high schools and a total number of 9,356 schoolchildren in this age-range enrolled.

It is important to emphasize that the PHC units are distributed in the less favored socioeconomic regions of the municipality. All public high schools (n = 21) in the territorial areas covered by the PHCs were enrolled. In the 34 PHC units, the Terms of Free and Informed Consent to participate in the Study were handed to the Community Health Agents during home visits. These agents also previously made the appointments for the time and day for participants to appear at the units. At the schools, the Terms were handed to the teachers who distributed them to the selected schoolchildren, in order to obtain the parents’ or guardians’ authorization afterwards.

### Sample

The sample size was calculated based on the caries experience in the Southeastern region of Brazil using data from a previous national epidemiological survey, considering a sampling error of 5%, DMFT = 5.16 with SD = 4.54, sample loss of 20% and a level of confidence 95%, obtaining a sample of 1,428 individuals aged 15 to 19 years, proportionally and randomly taken from 34 PHC-HF units areas existent in the municipality. The absentees candidates for selection were contacted and examined in the 21 public high schools.

Of these 1,428 adolescents initially selected, 249 failed to appear on the day of the exam or did not wish to participate. Thus, 221 individuals were examined at the 34 FHUs and 958 at the 21 state high schools, totaling 1,179 adolescents examined. Most of them had lived in the same suburb since their birth.

### Inclusion and exclusion criteria

The criteria for exclusion from the study were systemic diseases, difficulties with communication, or neuromotor problems, severe hypoplasia and orthodontic appliance. Individuals who did not agree to participate in the study and those absent on the day of the exam were excluded from the sample.

### Clinical examination

The exams were performed on the premises of the PHC-HF units and at the state high schools, by two examiners (previously calibrated and helped by two note-takers), in an outdoor setting, under artificial light using a flashlight and with brushing previously performed under the guidance of a Dental Assistant. For each exam, a ball point probe and plane oral mirror [27] were used. Data were collected with reference to the clinical characteristics: caries by the SiC index (Significant Caries Index) for one-third of the children with the highest caries scores [28] and by the DMFT index (total decayed, missing and filled teeth), periodontal disease (Community Periodontal Index-CIP), fluorosis (Dean Index) and use and need for dental prosthesis, in accordance with the World Health Organization [27] codes and criteria.

### Training and calibration

The process of calibrating the two examiners for the clinical conditions was conducted by a Gold Standard examiner. The theoretical-practical activities of the training and calibration exercises consisted of a total of 7 periods - 1 theoretical, lasting 4 hours, 4 clinical training sessions of 4 hours each (total of 16 h) and 2 calibration exercises lasting 4 hours (total of 8 h). The training stage consisted of a theoretical discussion, afterwards followed by a practical stage, in which the examiners evaluated 12 adolescents per period, with differentiated prevalence of oral diseases (caries, periodontal disease and fluorosis). Discussions were held between the examiners and the Gold Standard examiners with the purpose of obtaining an estimate of the extent and nature of the diagnosis, and up to this point the acceptable consistency, measured by KAPPA statistics, remained above 0.91 for all the clinical conditions [29]. The final calibration exercise consisted of 2 periods (total of 8 h) with mean inter-examiner Kappa values of 0.95. In order to verify maintenance of the diagnostic criteria and intra-examiner error, 10% of the sample were re-examined, showing a mean Kappa values of 0.96.

### Variables studied and instruments used

The individual and contextual variables analyzed are described in Table 1.

**Table 1:** **Individual and contextual variables**

Individual variables	Clinical Variables	Caries
		Periodontal Disease
		Fluorosis
	Sociodemographic and psychosocial variables of the adolescent and family	Age
		Sex
		Monthly Family Income
		Number of persons in the family
		Fathers’ and Mothers’ Educational Level
		Type of housing
		Family Grant Program
		Prison inmate in the family
		Has lived in a city other than Piracicaba
		Number of siblings
		Failure to pass end of year school tests
		Adolescent works
		Father and mother work
	Self-perception and Impact on Oral Health	How do you classify the health of your teeth and mouth?
		Are you satisfied with the appearance of your teeth
		OIDP
	Access to Services	Have you ever been to the dentist?
		What type of dental service do you generally use?
		When was the last time you went to the dentist?
		Why did you go to the dentist the last time?
		What is your most frequent reason for going to the dentist?
	Quality of Life	WHOQOL-BREF
Contextual variables	Information about suburbs	Social Exclusion Index
		Total number of residents per suburb
		Literacy Rate
		% of home ownership
		% domestic sewage facilities
		% garbage collected
		% with income up to 0.5 minimum wage
		% with income up to 1 minimum wage
		% with income over 1 minimum wage
		% without monthly income

Piracicaba, SP, Brazil, 2012.

### Individual variables

A self-administered questionnaire was applied, under supervision in case of doubt, to collect the sociodemographic variables [30] and another to obtain information on psychosocial, self-perception of health and access to health services variables [31].

The instruments used for evaluating quality of life and socio-dental impact were the WHOQOL-bref [32] and OIDP (Oral Impacts on Daily Performances) [33] respectively.

The Basic Care Information System (SIAB) is an instrument used by the Brazilian Primary Healthcare System to enroll the families in the territory covered by the PHC-FH units and records socio-sanitary data and those relative to the living conditions of these persons, with the aim of planning the interventions and health care [34]. By means SIAB it was possible to obtain information with reference to the adolescent’s family belonging to an income transfer program (Family Grant Program) [35] and whether there was any family member serving a prison sentence. Brazil instituted the Family Grant Program (FGP) in 2004, with the purpose of transferring income directly to families in a situation of poverty and extreme poverty, and guarantee the right to basic social services, making it possible to benefit 16 million Brazilians (8.5% of the population) [36].

### Contextual variables

The Social Exclusion Index (SEI) of the 36 suburb where the adolescents resided was collected at the Piracicaba Research and Planning Institute and the Municipal Secretary for Social Development [37]. The purpose was the quantitative dimensioning of some of the attributes of social inequalities between the suburbs, ranging from -1 (suburbs with the worst indices – most vulnerable) to 1 (suburbs with the best indices – least vulnerable).

Other items of information with reference to suburbs where the adolescents resided (total number of residents per suburb, literacy rate, % of home ownership, % domestic sewage available, % garbage collected, % with income of up to 0.5 minimum wage, % with income from 0.5 to 1 minimum wage, % with income higher than 1 minimum wage and % without monthly income) were obtained from the Brazilian Institute of Geography and Statistics [38].

### Data analysis

In the present study, the “number of decayed teeth” and the “DMFT index” were considered dependent variables.

Multilevel regression models were estimated by the PROC GLIMMIX - “Generalized Linear Models-Mixed” procedure using the SAS 9.2, statistical software program (SAS/STAT Guide for personal computers. Cary: North-Carolina/USA; 2001). In the analysis, the individual variables were considered as being level 1 and those of the suburbs, as being level 2. Adjustment of the model was evaluated by -2 Res Log Likelihood (the lower, the better fit the model).

Initially a model was estimated only with the intercept, in order to study the proportion of variance due to the suburbs in relation to the individuals. This model served as a basis for evaluating the reduction in variance in the other model studied (Model 1). After this, the individual variables were tested (Model 2) and then those of the suburbs were included (Model 3).

## Results

The response rate in this study was 82.6%. The descriptive analysis (frequency and percentage) of some independent variables is shown in Table 2.

**Table 2:** **Sample description**

Individual variables	n	%
**Sex**		
Female	659	55.89
Male	520	44.11
**Age (years)**		
15	815	69.13
16	231	19.59
17	82	6.96
18	29	2.46
19	22	1.87
**Number of persons in the family**		
Up to 2	31	2.65
3	161	13.78
4	346	29.62
5	300	25.68
6	173	14.81
Over 6	157	13.44
**Has a family member in prison**		
No	1141	99.30
Yes	8	0.70
**How would you classify the health of your teeth**		
Excellent	69	5.88
Very good	229	19.51
Good	468	39.86
More or Less	363	30.92
Poor	45	3.83
**When was the last time you went to the dentist**		
I am undergoing treatment at present	274	24.89
Less than 6 months ago	329	29.88
7-12 months ago	158	14.35
13-24 months ago	52	4.72
Over 24 months ago	83	7.54
Don’t know/Don’t remember	205	18.62
**Monthly family income**		
Up to 1 minimum wage*	106	9.18
Over 1 up to 2 minimum wages	304	26.32
Over 2 up to 3 minimum wages	280	24.24
Over 3 up to 5 minimum wages	278	24.07
Over 5 up to 7 minimum wages	102	8.83
Over 7 up to 10 minimum wages	63	5.45
Higher than 10 minimum wages	22	1.90
**Last visit to the dentist**		
I am in treatment at the time	274	24.89
Less than 6 months	329	29.89
7-12 months ago	158	14.35
13-24 months ago	52	4.71
More than 24 months	83	7.54
Do not remember	205	18.62
**Contextual variable**	n	%
**Social exclusion index**		
-1 to -0.75	322	27.31
-0.74 to -0.5	267	22.65
-0.4 to -0.25	491	41.64
-0.24 to 0	60	5.10
0.1 to 0.25	24	2.03
0.26 to 0.5	12	1.02
0.6 to 0.75	3	0.25

*Minimum wage at the time of the data collection ≅ US$ 320.00.

Piracicaba, SP, Brazil, 2012.

Of the 1,179 study subjects, 1080 (91.6%) resided in suburbs with the worst social exclusion indices. The median, minimum and maximum value for the variable related to % of domestic sewage was 99.49, 84.89 and 100 respectively. The larger portion of the sample were 15 years of age (69.13%). There was a balance between those examined as regards sex, with 55.89% being female, and 39.86% classified the health of their teeth and mouth as being good. As regards monthly Family income, the larger portion of the volunteers came from families with an income ranged from 2 to 5 minimum wages.

With regard to the clinical variables, the mean (SD) DMFT index was 2.10 (2.71), with the decayed, missing and filled component being a mean value (SD) of 0.47 (1.05), 0.09 (0.48) and 1.53 (2.32), respectively. The DMFT for the high caries-level individual (polarization group) presented a SiC index of 5.24. Moreover, the majority of the adolescents examined (95.42%) did not need dental prostheses.

Table 3 shows the results of the multilevel analysis for the number of decayed teeth (dependent variable). By means of Model 1, the intraclass correlation coefficient r = 0.0132/(0.0132 + 1.0859) = 0.0120 was obtained; that is, the variation in the number of decayed teeth between the suburbs represented approximately 1% of the total variation.

**Table 3:** **Multilevel model for number of decayed teeth**

Variable	Model 1			Model 2			Model 3		
	Estimate	SE	p-value	Estimate	SE	p-value	Estimate	SE	p-value
Intercept	0.4407	0.0397	<0.0001	0.5673	0.3791	0.1420	0.6717	0.3823	0.0864
Individual level									
Prison inmate in the family (ref = yes)				-1.0167	0.3513	0.0039	-1.0214	0.3509	0.0037
Number of persons in the family				0.0686	0.0227	0.0026	0.0721	0.0227	0.0015
Health of teeth and mouth				0.2079	0.0314	<0.0001	0.2118	0.0314	<0.0001
Suburb level									
Social exclusion index (SEI)							0.2392	0.1145	0.0369
Variances									
Between suburbs	0.0132	0.0092		0.0078	0.0074		0.0039	0.0063	
Between individuals	1.0859	0.0450		0.9682	0.0410		0.9684	0.0410	
2 Res log likelihood	3457.25			3209.32			3207.85		

Piracicaba, SP, Brazil, 2012.

When the individual variables were included in Model 2, the reduction in 2 Res Log Likelihood was approximately 7%. When the variable “suburbs” - significant in Model 3 - was included, the reduction in comparison with the previous model was approximately 0.04%, which confirmed that the variation in the number of decayed teeth, due to the variables related to the individuals, was more important than that related to the suburbs.

Considering the level of significance of 5%, by Model 3, it could be affirmed that the volunteers whose family had a relative in prison, presented a higher number of decayed teeth (p = 0.0037) than those who did not have one. With regard to number of persons in the family household, the adolescents who lived in residences with a larger number of persons also presented a higher number of decayed teeth (p = 0.0015).

An increase in the number of decayed teeth was also found in adolescents with the worst self-perception of the health of their teeth and mouth (p < 0.0001).

In addition to the variables relative to the individuals, in Model 3, one observed that there was an increase in the number of decayed teeth as the social exclusion index worsened (p = 0.0369). The other variables did not have a significant influence on the number of decayed teeth (p > 0.05).

Table 4 shows the results of the multilevel analysis for the DMFT caries Index. By means of Model 1, the intraclass correlation coefficient r = 0.1417/(0.1417 + 7.1852) = 0.01934 was verified; that is, the variation in DMFT between the suburbs represented approximately 2% of the total variation. When the individual variables were included in Model 2, the reduction in 2 Res Log Likelihood was approximately 9%. When the variable “suburbs” - significant in Model 3 -was included, the reduction in comparison with the previous model was approximately 0.003%, which confirms that the variation DMFT due to the variables related to the individuals is more important than that related to the suburbs.

**Table 4:** **Multilevel model for the caries index (DMFT)**

Variable	Model 1			Model 2			Model 3		
	Estimate	SE	p-value	Estimate	SE	p-value	Estimate	SE	p-value
Intercept	2.0729	0.1129	<0.0001	4.1380	1.2125	0.0014	2.4722	2.6456	0.3555
Individual Level									
Sex (ref = male)				0.5515	0.1641	0.0008	0.5629	0.1635	0.0006
Age (years)				0.3436	0.0779	<0.0001	0.3305	0.0777	<0.0001
Health of teeth and mouth				0.4984	0.0884	<0.0001	0.5072	0.0881	<0.0001
Last visit to the dentist				-0.2598	0.0456	<0.0001	-0.2571	0.0455	<0.0001
Suburb level									
% of domestic sewage							-0.0656	0.0232	0.0048
Variances									
Between suburbs	0.1417	0.0844		0.0645	0.0600		0.0000	-	
Between individuals	7.1852	0.2986		6.9196	0.3009		6.9371	0.2994	
2 Res log likelihood	5687.77			5172.32			5172.15		

Piracicaba, SP, Brazil, 2012.

Considering the level of significance of 5%, in Model 3, it was observed that the volunteers of the female gender presented a higher DMFT than those of the male gender (p = 0.0006).

The DMFT increased with age (p < 0.0001) and diminished when the time since the last visit to the dentist increased (p < 0.0001). The increase in DMFT also drew attention when the adolescents’ self-perception with regard to the health of the teeth and mouth (p < 0.0001) was classified as worse. In addition to these variables with reference to the individuals, in Model 3 it was observed that the DMFT diminished slightly, but significantly (p = 0.0048) with the increase in the percentage of domestic sewage in the place where the volunteers lived.

## Discussion

First of all, there is a body of evidence in the literature demonstrating the association between income inequalities and caries prevalence [9–12]. In the present study, we also found association between socioeconomic variables and the number of decayed teeth. Traditionally, SES and monthly family income have been used in epidemiological studies as predictor variables and both play important roles in the modulation of the health/disease process [5, 39]. Nevertheless, these variables are very sensitive when we study socioeconomically leveled populations. Therefore, other variables appear to better explain the relationship between caries and social inequalities especially in underprivileged groups. This study is probably the first to find “Prisoner in Family” as a variable associated with a higher number of decayed teeth in adolescents. In studies conducted with the purpose of analyzing children with an imprisoned relative, it is important to understand their emotional needs and vulnerabilities related to mental health problems (2.5 time more chances of experiencing mental problems than other children do), in addition to having greater social disadvantages and being victims of other negligence by the protection system [40]. Furthermore, these individuals are among the socially less favored, and the imprisonment of some family member aggravates their privation even further, so that they need to struggle every day to deal with debt, poverty, loneliness, ostracism, stigma and lack of housing [41].

The number of persons in the family household has been associated with caries, healthy nutritional and hygiene habits, oral health-related quality of life, traumatic dental injuries in children and adolescents [19, 42]. In addition to a relationship of risk, this variable clearly expresses social vulnerability and acts as a strong explanatory variable, and there is a relationship of family environment and socioeconomic status (SES) and oral health conditions [12]. This appears to be even clearer when we verify that the sample comes from peripheral areas where there is a concentration of adolescents in low SES. Therefore, an important finding of this study is that in spite the significant decrease in caries prevalence observed over the last 3 decades in Brazil [2], one has to be aware of its distribution in the population. In the city of present study, we detected that there was remarkable inequality in this distribution, even among the less favored adolescents, since the variable “social exclusion index of the suburbs” was significant in the multilevel model for the number of decayed teeth. This affirmation became evident when we verified that the mean value of the DMFT index of the group was 2.1, however, caries experienced increased to 5.24 (SIC) in the group in which high levels of the disease were concentrated.

We were able to find a relationship between individual variables and both dependent variables: number of decayed teeth (perception of teeth and oral conditions) and DMFT (gender, perception of teeth and oral conditions, dental service use, adolescent’s age).

Dental services use was positively associated with DMFT, in a manner similar to findings of other studies from developing countries [43, 44]. The most plausible explanation for this fact in our study is that the adolescents are under the care of oral health teams, which allows greater access to public dental treatment at the PHC-FH units. The Filled component accounts for 72.9% of DMFT index. However, in developed countries there is an inversely proportional relationship between SES and use of dental services by adolescents [8]. The explanation for this is based on the fact that adolescents of the more privileged classes have a lower prevalence of oral health problems in those countries, and therefore, present a lower demand for service.

Health services are important causal pathways to oral health outcomes [7, 45, 46] and can explain socioeconomic disparities in oral health [47]. Dental services generally do not deal with the social determinants of health with implications for oral diseases. However, it is known that they have important impact on health inequalities when they improve accessibility and respond appropriately to the healthcare needs of different social groups [42, 48].

Our results showed that girls presented more caries experience (DMFT) than boys. Evidences have shown that girls take better care of their teeth and go to the dentist more frequently [49]. This is related to the better care taken of health and esthetic appearance of teeth [11]. However, our findings may be explained in two ways: greater access to dental care results in an increase in the DMFT index, especially restored teeth, frequently the result of overtreatment, and Brazilian adolescent girls, who were never poor in their trajectory of life, presented higher DMFT [11].

Another expected and significant variable related to DMFT was the age of adolescents. Our study was developed in high schools, including all the schoolchildren enrolled in the 10th and 11th grades. Therefore, there was a greater probability that we would find adolescents of 15 and 16 years.

Perception of one’s own teeth and impact of oral health on daily activities and on oral conditions is an important measurement of health, notwithstanding the fact that traditional measures have been based on objective variables [8]. In the present study, the relationship between worse self perception of teeth and oral condition and higher number of decayed teeth was observed, as in other studies [8, 19, 50, 51] that have reported intervenient variables such as the use of health services, daily tooth brushing and income.

We found that there was a relationship between contextual variables and the number of decayed teeth (social exclusion index of the suburb) and DMFT (% of residences in the suburbs that have sewage treatment). Authors have argued that there is enough evidence to affirm that there is a link between individual and socioeconomic variables of the areas in which the residents live, and dental caries [15, 52]. A lower percentage of adolescents with toothache was found in a Brazilian suburb with higher social capital [53], as well neighborhood empowerment and dental caries [52, 54, 55]. Furthermore, social capital appears to be related to self-esteem, social support and parents’ political interest in adolescents [52, 56]. Possibly, the negative effects of stress may be greater for residents in disadvantaged neighborhoods with the least social support and fewest network resources [24, 57], resulting the worst level of health status. However, most of the variance in DMFT occurred at the individual level and only 2% of the variance occurred at the community level, a result similar to that found by Tellez et al. [54] but lower than the findings of Aida [55]. The difference can be explained by the sample design used in each study, especially population characteristics and sample size. Nevertheless, one of the important findings is that contextual variables must be considered by health managers in health planning, in order to seek greater equity in the distribution of health services and to target the reduction of social inequalities.

One of the differentials of this study was the use of a multilevel model in the analysis of factors related to dental caries in adolescents. Multilevel Models are appropriate for the analysis of hierarchical data, considering the influence of community context on the health behaviors of individuals with regard to caries experience [52, 55, 58]. Furthermore, we used a recent technique (PROC GLIMMIX) which presents better estimates and gives substantive meanings to data clustering in comparison with traditional regression analysis, allowing contextual factors to be analyzed separately [24]. The modeling of oral health data is rather complex, since these data generally do not present normal distribution. With the development of generalized linear models (an extension of linear models for non-normally distributed data) this type of problem has been significantly reduced. Whereas mixed generalized linear models incorporate the random effects in the predictor, thus being most useful in data with superdispersion. The application of mixed generalized linear models has been satisfactorily used in multilevel analysis [24, 59]. Possibly, this study is one of the first to use this technique for studying the relationship of individual and contextual variables with regard oral health.

Lastly, our study has some limitations. This was a cross-sectional study and sought inferences with regard to causal factors without, however, establishing a temporal relationship. The socioeconomic information was obtained from the adolescents and their parents, which of itself could be a source of response bias. Furthermore, no individual variables related to behavioral (dietary practices and oral hygiene habits) as well as bacterial and salivary factors were collected. The self perception questionnaire may have been influenced by social acceptance and social desirability [8, 55]. Moreover, obtaining data about income is very sensitive, and the respondents may not have informed accurately [8]. Finally, fluoride has possibly has a beneficial effect on reducing caries in this population of adolescents and a reduction of inequality can also be expected when compared with the same population not supplied with fluoridated water [3].

There was a considerable variation in caries that could not be explained by the individual and contextual variables assessed. This is understandable because there are many variables (contextual, individual, behavioral and others) in the process of developing caries, which are not always controlled in a statistical model.

## Conclusion

The variations in the DMFT and the number of decayed teeth in the sample were mainly related to individual variables. However, the context in which the individual lives was measured by an index of social exclusion, was shown to be relevant even in underprivileged individuals.
